# Tuning the interactions between electron spins in fullerene-based triad systems

**DOI:** 10.3762/bjoc.10.31

**Published:** 2014-02-05

**Authors:** Maria A Lebedeva, Thomas W Chamberlain, E Stephen Davies, Bradley E Thomas, Martin Schröder, Andrei N Khlobystov

**Affiliations:** 1 School of Chemistry, University of Nottingham, Nottingham, NG7 2RD, UK; 2 Nottingham Nanoscience & Nanotechnology Centre, University of Nottingham, University Park, Nottingham, NG7 2RD, UK

**Keywords:** carbon nanomaterials, electrochemistry, EPR, fullerene dimers, fullerene triads, spin–spin interactions

## Abstract

A series of six fullerene–linker–fullerene triads have been prepared by the stepwise addition of the fullerene cages to bridging moieties thus allowing the systematic variation of fullerene cage (C_60_ or C_70_) and linker (oxalate, acetate or terephthalate) and enabling precise control over the inter-fullerene separation. The fullerene triads exhibit good solubility in common organic solvents, have linear geometries and are diastereomerically pure. Cyclic voltammetric measurements demonstrate the excellent electron accepting capacity of all triads, with up to 6 electrons taken up per molecule in the potential range between −2.3 and 0.2 V (vs Fc^+^/Fc). No significant electronic interactions between fullerene cages are observed in the ground state indicating that the individual properties of each C_60_ or C_70_ cage are retained within the triads. The electron–electron interactions in the electrochemically generated dianions of these triads, with one electron per fullerene cage were studied by EPR spectroscopy. The nature of electron–electron coupling observed at 77 K can be described as an equilibrium between doublet and triplet state biradicals which depends on the inter-fullerene spacing. The shorter oxalate-bridged triads exhibit stronger spin–spin coupling with triplet character, while in the longer terephthalate-bridged triads the intramolecular spin–spin coupling is significantly reduced.

## Introduction

Fabricating molecular systems that are capable of storing one or more unpaired electrons is essential for the development of molecular spintronics and electron-spin-based quantum computing. Endohedral fullerenes are compounds that contain a heteroatom trapped inside the fullerene cage and are able to support the formation of stable radical materials [1]. They can also show interesting properties such as magnetism, and photoactivity and are thermally and chemically stable. Their ability to form well-ordered 1D arrays makes them leading candidate materials for the study of polyfunctional materials. For example, significant effort has been directed into incorporating N@C_60_ molecules into quantum computing devices [2]. Incorporating a second radical centre into these molecules, in addition to the endohedral atom, introduces a mechanism to control the magnetic properties of the resulting materials. This is essential for the recording, storing and read-out processes performed in spin-based quantum information processing using nanoscale molecular architectures [3]. This has been achieved recently within a copper porphyrin–N@C_60_ dyad [4] and several types of N@C_60_–N@C_60_ molecules [5–7]. However the application of these systems is limited due to a number of synthetic challenges associated with the preparation and purification of endohedral fullerenes [8]. This notwithstanding, fullerene cages are excellent electron acceptors and can support up to six electrons per fullerene cage to form species containing one or more unpaired electrons, in which overall charge and the spin state can be controlled precisely by applied potential [9]. In addition, combining two fullerene cages within the same molecule increases the total spin-carrying capacity and introduces a mechanism of spin-tuning while retaining the intrinsic properties of each of the fullerene cages [10]. The synthesis of such fullerene–bridge–fullerene triads, though not straightforward, has been reported [11], the simplest involving species where the fullerene cages are connected directly by a C–C bond [12], a bridging O-atom [13], or by a transition metal atom [14]. A variety of more complex triads have since evolved in which the fullerene molecules are connected using optically or electrochemically active spacers [15]. The choice of linker in triad systems is crucial as it has a significant impact on the properties of the resulting arrays [16]. As the strength of dipolar coupling between unpaired electrons decreases as a function of *r*^−3^, where *r* is the average distance between unpaired electrons, the strength of any electron–electron interactions in fullerene triads rapidly decreases with increasing distance between fullerene cages [17]. Thus, the ability to control the inter-fullerene separation is crucial in fabricating systems in which specific interactions between multiple unpaired electrons are targeted.

The shape of the fullerene containing molecule is also very important. 1D and 2D ordering is a critical factor in the design of molecular electronics. For example, linear molecules can be ordered readily into 1D arrays using carbon nanotubes as templates [18] and are therefore advantageous compared to non-linear or branched molecules for which 1D packing arrangements are inhibited. In addition, solubility can also be a significant issue as fullerene triads tend to show poor solubility [19]. The majority of fullerene triads reported to date are either synthesised via complicated non-scalable synthetic procedures, which makes controlling the fullerene–fullerene distance difficult, or incorporate bulky spacers and solubilising groups resulting in cumbersome non-linear structures and hence are not ideal for potential applications in molecular electronics and spintronics devices. We recently reported a new general synthetic methodology for the formation of fullerene triads which allows the introduction of fullerene cages in a stepwise fashion and thus allows the length of the spacer to be adjusted [20]. We report herein the preparation of six different fullerene–linker–fullerene triads in which both the length of the linker and the nature of the fullerene cage are systematically varied, and we explore their spin-carrying and spin-tuning capacity in reduced states using electrochemical techniques and electron paramagnetic resonance (EPR) spectroscopy.

## Results and Discussion

### Synthesis of the fullerene triads

**Figure 1:**
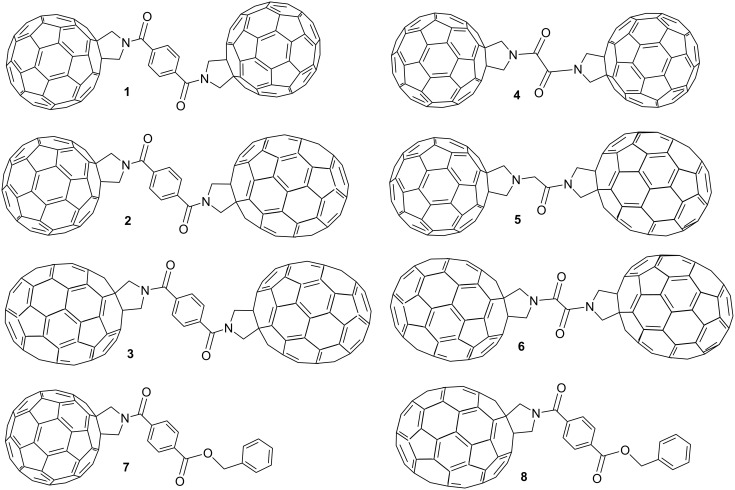
Structures of triads **1**–**6** and precursor molecules **7**–**8** used for the synthesis of the asymmetric systems. For the C_70_ containing compounds only the major (8,25) regioisomer is shown for clarity.

This study aimed to vary the fullerene–fullerene separation and the nature of the fullerene cages resulting in the preparation of six fullerene–linker–fullerene (triad) compounds (Figure 1). The fullerenes were functionalised via Prato reaction chemistry forming a pyrrolidine ring across the [6.6] bond of the cage [21]. The resulting pyrrolidine functionalised fullerenes are known to be electrochemically stable and can be readily linked together via the N atom to form linear and diastereomerically pure triads. The choice of linker was determined by the target fullerene–fullerene separation in the product, and terephthalate and oxalate bridges were chosen as they possess similar chemical properties but differ significantly in size. The distance between the centres of the corresponding fullerene cages in the terephthalate bridged triads (compounds **1**–**3**) was estimated to be 16–20 Å depending on the conformation of the molecule (see Figure S4, Supporting Information File 1), whereas the oxalate (compounds **4** and **6**) or acetate (compound **5**) bridged triads have significantly shorter separations (12–15 Å). Functionalised fullerenes **7** and **8**, which are precursors in the synthesis of triads **1**–**3**, were utilised as control compounds in the electrochemical studies and to aid the assignment of redox processes in the triad species.

**Scheme 1:**
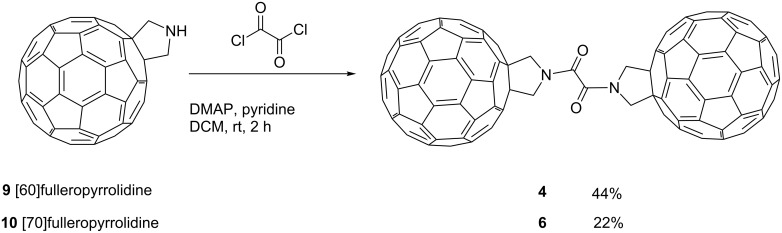
The one-step synthetic procedure towards the oxalate-bridged fullerene triads **4** and **6**.

The triads **1**–**3** were synthesised in five steps by functionalisation of each fullerene cage using the Prato reaction, addition of the terephthalate spacer to the fulleropyrrolidine unit and subsequent coupling of two fullerene moieties [20]. The oxalate or acetate bridged triads **4**–**6** were prepared using a similar strategy. To link the two C_60_ or two C_70_ fullerene cages with the oxalate spacer (compounds **4** and **6**) a one-step procedure was used in which the corresponding fulleropyrrolidine was treated with an excess of oxalyl chloride in the presence of 4-dimethylaminopyridine (DMAP) and pyridine (Scheme 1).

Oxalate bridged triads **4** and **6** were obtained in moderate yields and displayed physical and spectroscopic properties very similar to those of terephthalate bridged triads **1**–**3**, including good solubility in organic solvents such as CS_2_ and *o*-dichlorobenzene (ODCB).

**Scheme 2:**
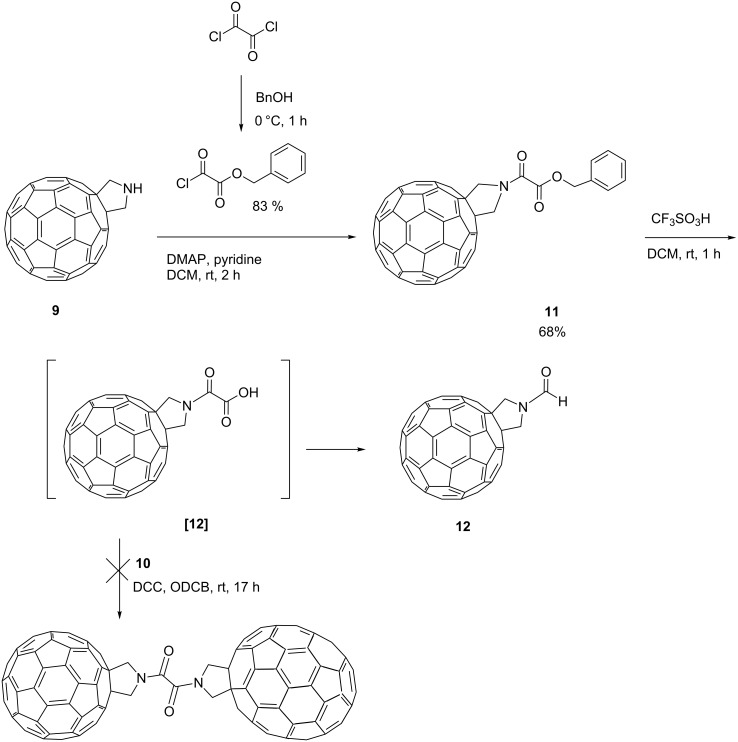
Attempted synthetic pathway towards the formation of the C_60_–C_70_ oxalate bridged fullerene triad allowing the coupling of the fullerene cages in a stepwise fashion.

To link the C_60_ and C_70_ fulleropyrrolidines within asymmetric triads with an oxalate spacer we attempted a similar stepwise approach as reported for compounds **1**–**3** (Scheme 2).

Oxalic acid monobenzyl ester monochloride was prepared by equimolar reaction of oxalyl chloride and benzyl alcohol [22] and was reacted with [60]fulleropyrrolidine **9** to give the benzyl ester protected compound **11** in 68% yield. Subsequent deprotection of **11** by CF_3_SO_3_H yielded the insoluble product **12** that precluded characterisation by solution based methods. However, MALDI–MS of **12** showed a molecular ion peak with *m*/*z* 791 and the solid state IR spectrum as a pressed disc in KBr indicated only one signal in the carbonyl region characteristic of the amide group (1653 cm^−1^) and no signal related to the carboxylic group stretch. The desired carboxylic acid compound [**12**] seems to be unstable under acidic conditions and undergoes decarboxylation to form an insoluble amide compound **12**. This reaction under acidic conditions is characteristic of carboxylic acids that contain an electron withdrawing substituent in the α-position [23].

**Scheme 3:**
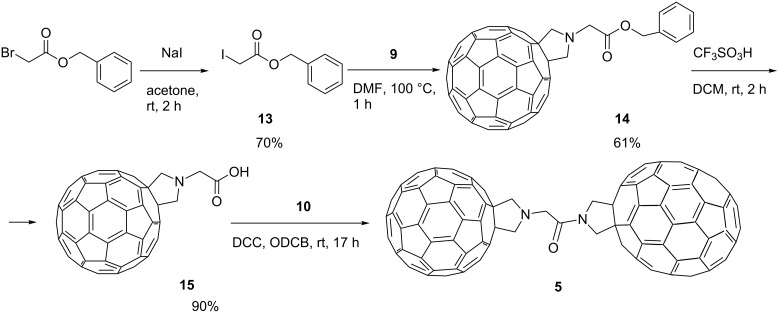
Synthetic pathway to the asymmetric fullerene triad **5** allowing introduction of the fullerene cages in a stepwise fashion.

To prevent decarboxylation processes we modified the linker to exclude the electron withdrawing amide group from the α-position of the carboxylate functionality, while maintaining an identical inter-fullerene separation in the asymmetric triad when compared with the symmetric analogue (Scheme 3).

The key step in this procedure is the formation of intermediate compound **14** via the nucleophilic substitution of the iodide centre in benzyl 2-iodoacetate [24] (**13**) with [60]fulleropyrrolidine **9** (Scheme 3). Compound **14** is a benzyl ester of an α-amino acid which is stable under acidic conditions. Indeed, deprotection of **14** yielded the desired carboxylic acid **15**. Compound **15** also shows limited solubility, but MALDI–MS (*m*/*z* 821) and IR spectroscopy (carbonyl stretch at 1733 cm^−1^) confirm the assigned structure. The dicyclohexylcarbodiimide (DCC) assisted acid–amine coupling reaction between **15** and [70]fulleropyrrolidine **10** resulted in the formation of the desired asymmetric triad **5**, which displayed physical properties similar to those of **1–4** and **6**.

### Electrochemical characterisation of fullerene triads **1**–**6**

Compounds **1**–**6** were studied by cyclic voltammetry (CV) as solutions in *o*-dichlorobenzene in order to investigate the sequence of electron additions and possible electronic interactions in these triads.

**Figure 2:**
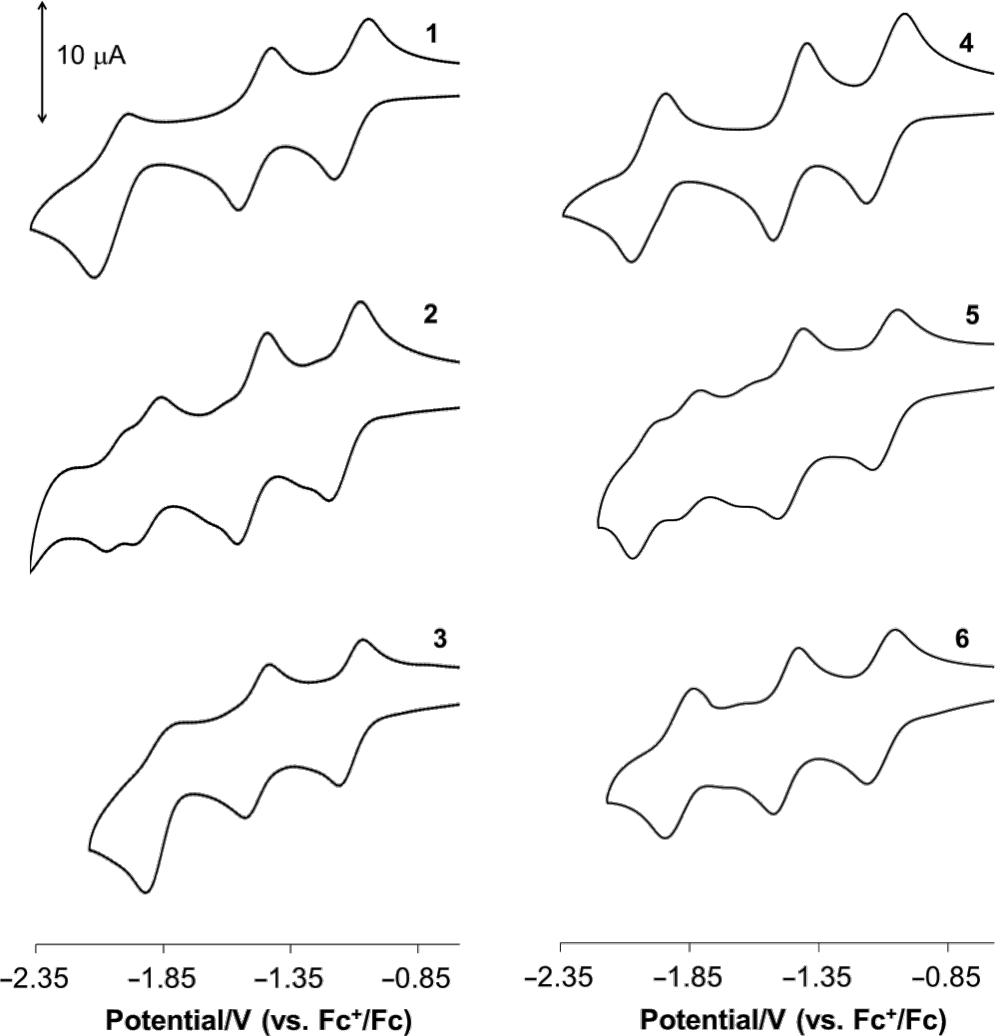
Cyclic voltammograms of the terephthalate bridged triads **1–3** (left) and oxalate bridged triads **4–6** (right). Data were recorded as 0.5 mM solutions in *o*-dichlorobenzene containing 0.2 M [*n*-Bu_4_N][BF_4_] as the supporting electrolyte, at a scan rate of 100 mV.

The cyclic voltammograms recorded for the fullerene triads **1**–**6** are very similar to those of their monomeric precursors **7** and **8** and do not appear to show interactions between the individual fullerene cages. This observation is consistent with the lack of an effective electronic communication pathway through the linker groups within the triads (Table 1 and Figure 2).

**Table 1:** Electrochemical data^a^ for fullerene based compounds **1**–**8**.

Compound	*E*_1/2_ red_1_, V	*E*_1/2_ red_2_, V	*E*_1/2_ red_3_, V	Δ*E*, Fc^+^/Fc

**1**	−1.09 (0.16)	−1.47 (0.16)	−2.01 (0.16)	0.17
**2**	−1.12 (0.10)	−1.48 (0.08)	−1.89 (0.05) −2.02 (0.06)	0.20
**3**	−1.12 (0.06)	−1.50 (0.08)	−1.89 (0.06)	0.10
**4**	−1.09 (0.16)	−1.47 (0.16)	−2.01 (0.18)	0.16
**5**	−1.13 (0.10)	−1.49 (0.10)	−1.89 (0.11) −2.05 (0.10)	0.10
**6**	−1.10 (0.11)	−1.47 (0.10)	−1.89 (0.11)	0.12
**7**	−1.11 (0.09)	−1.49 (0.09)	−2.02 (0.08)	0.15
**8**	−1.15 (0.06)	−1.52 (0.06)	−1.94 (0.06)	0.09

^a^Potentials (*E*_1/2_ = (*E*_p_^a^ + *E*_p_^c^)/2) in volt are quoted to the nearest 0.01 V. All potentials are reported against the Fc^+^/Fc couple for 0.5 mM solutions in *o*-dichlorobenzene containing 0.2 M [*n*-Bu_4_N][BF_4_] as the supporting electrolyte. The anodic/cathodic peak separation (Δ*E* = *E*_p_^a^ − *E*_p_^c^) is given in brackets where applicable. Δ*E* for the Fc^+^/Fc couple was used as the internal standard.

Each triad molecule **1**–**6** exhibits a series of reduction processes in a potential range between −0.3 and −2.3 V (vs Fc^+^/Fc), some of which are overlapping or appear as shoulders to the main peaks. By comparison with the cyclic voltammetry of the precursor compounds **7** and **8** (Figure S5, Supporting Information File 1), we suggest that this series of reductions corresponds to the addition of up to 3 electrons per fullerene cage (in total 6 electrons per molecule). No oxidation processes were found in the range up to 1.5 V (vs Fc^+^/Fc). For triads **1** and **4**, containing C_60_ only, these potentials are similar to those of their precursor **7**. A similar correlation was noted for the C_70_ containing triads **3** and **6** and their precursor **8**. Comparison of **1** with **3**, **4** with **6** and **7** with **8** shows that the first and second reductions of C_60_, in general, occur at slightly more anodic potentials than those of C_70_ cages. However beyond the second reduction, C_70_ is more readily reduced. For **2** and **5**, each containing a mixture of C_60_ and C_70_ fullerenes, two well defined reduction couples are observed at *E*_1/2_ ca. −1.12 and −1.49 V, which we assigned to an overlap of C_60_/C_70_-based reductions. These processes are separated by an additional process that appears as a shoulder on the first reduction in **2** and **5** (Figure 2 and Figure S6, Supporting Information File 1). A similar feature is noted to cathodic potential of the second reduction process. For **2** and **5** we associate these features with the generation of a reduced C_70_ cage in a triad molecule, noting a similar, although less pronounced effect in **3** and **6**, each containing two equivalent C_70_ cages (see Supporting Information File 1) and an absence of these features in C_60_ triads, **1** and **4**, and in the C_70_ containing dyad, **8**. The origin of these effects is unclear and may result from the nature of interaction of the reduced triad with the electrode surface. We note that the first and second reductions on C_70_ are expected to be slightly more cathodic than those for C_60_ but comparing potentials for **7** and **8**, we suggest that this difference alone is too small to explain the position of these features. We note also that **2** and **5** are mixtures of two regioisomers of the pyrrolidine functionalised C_70_ [19], in a ratio of 6:4 as determined by ^1^H NMR spectroscopy (see Experimental section). It is possible that these isomers may interact with the electrode differently.

Based on these results we can confirm that for C_60_–C_60_ triad molecules changing the nature and the size of the bridging group has little effect on the nature and potentials of the redox processes. Thus, we conclude that the two C_60_-fullerene cages in the triads behave independently in the ground state. These results are consistent with other fullerene triad systems in which intramolecular fullerene–fullerene interactions are only observed where fullerene cages are bonded directly [25] or bridged by a transition metal atom [26–27]. For triads containing C_70_ the results are less clear where additional electrode processes are observed. However we do not attribute these features to intramolecular fullerene–fullerene interactions.

### EPR spectroscopic characterisation of the fullerene triads in the reduced state

The electron spin–spin interactions that are crucial for the application of fullerene triads were investigated by EPR spectroscopy as fluid and frozen solutions at room temperature and 77 K, respectively, following electrochemical reduction. Whilst these triads are capable of accepting multiple electrons into each of the fullerene groups, we restrict our discussion to dianionic species in compounds where the electrochemistry is well defined; under these conditions each fullerene cage is reduced by a single electron. We have evaluated the effects of varying the inter-fullerene separation (oxalate bridge vs terephthalate bridge) and the nature of the fullerene (C_60_ vs C_70_) on the nature of the spin–spin coupling obtained.

The two electron reduced species of **1** and **4** (**1**^2−^ and **4**^2−^) and the corresponding mono reduced species of their monomeric precursor **7** (**7**^1−^) (i.e. one electron per fullerene cage for all species) were obtained by electrochemical reduction at −1.4 V of 0.5 mM solutions of compound in *o*-dichlorobenzene containing [*n*-Bu_4_N][BF_4_] as the supporting electrolyte.

**Figure 3:**
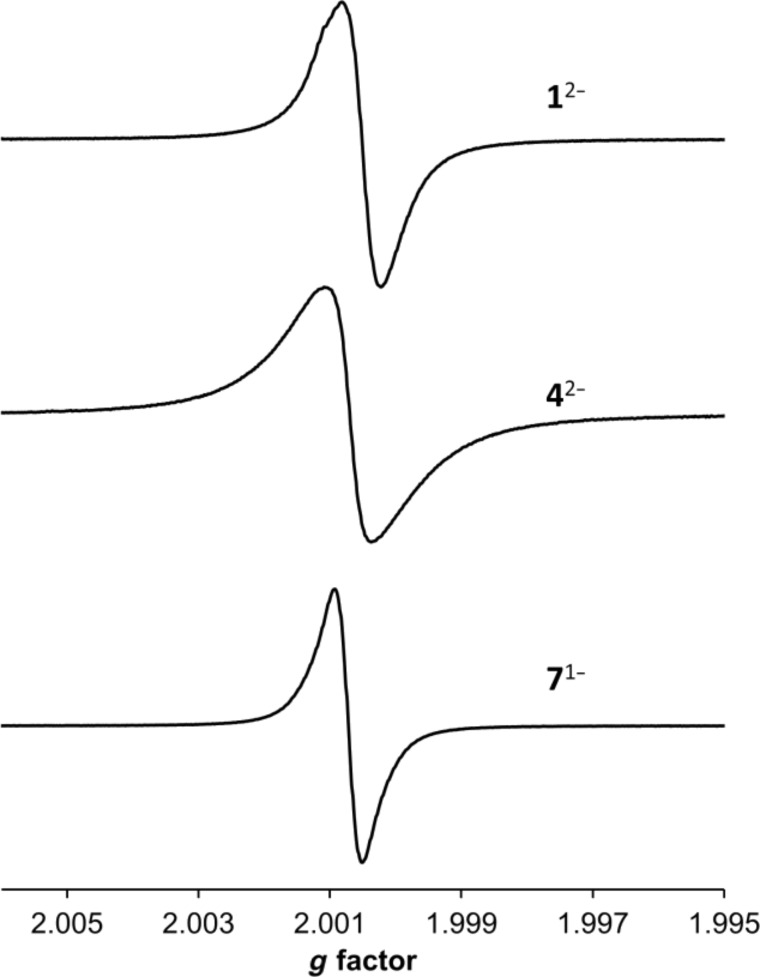
Fluid solution EPR spectra recorded at 297 K for the two electron reduced species of compounds **1** and **4** and the one electron reduced species of **7**.

Fluid solution EPR spectra of **1**^2−^, **4**^2−^ and **7**^1−^ (Figure 3) are similar in *g* value (2.0002, 2.0001 and 2.0000, respectively) but differ in linewidth (Δ*H*_p−p_ 1.1, 1.3 and 0.8 G, respectively) and are typical of C_60_ based radical anions [28], confirming that the electrochemically introduced electrons are localised on the fullerene cages.

**Figure 4:**
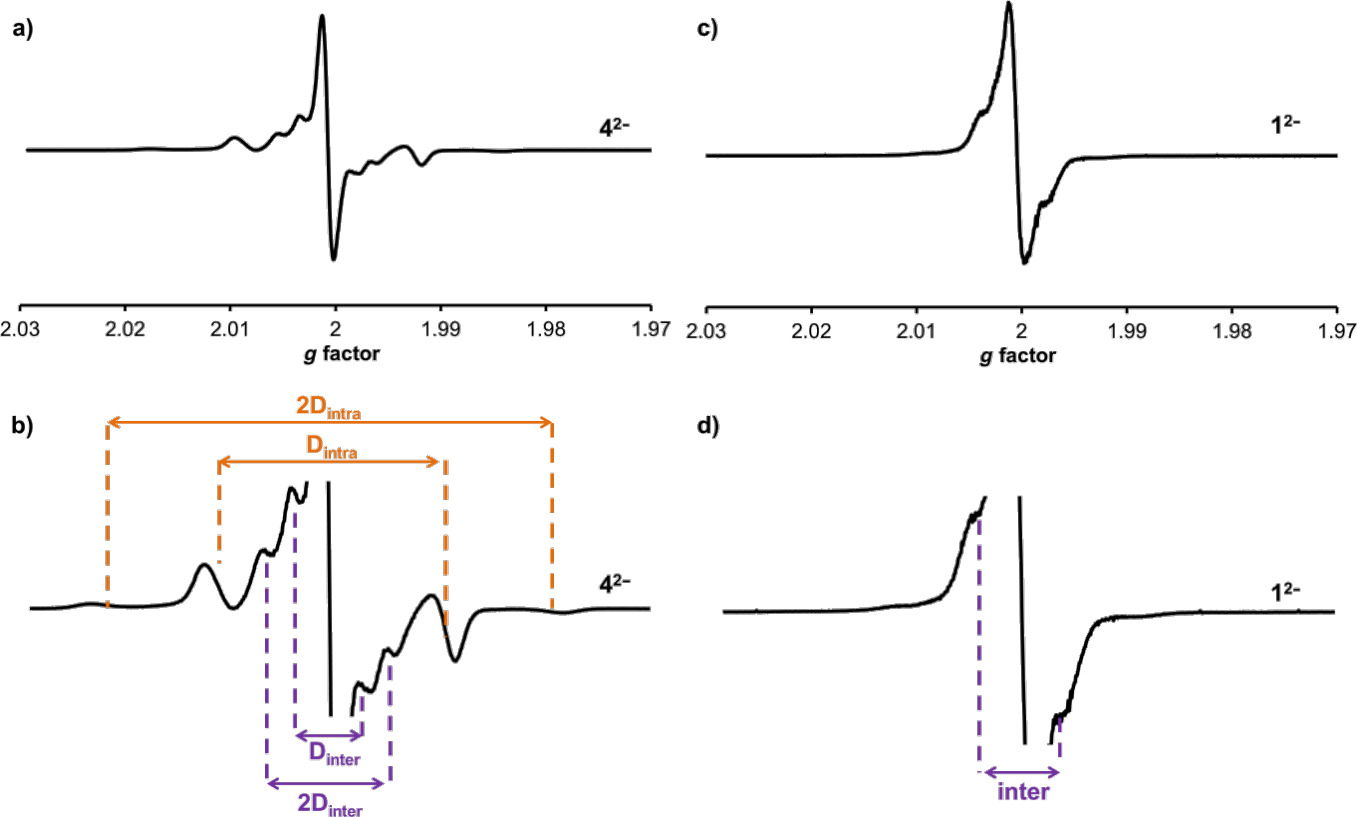
Frozen solution EPR spectra of triads **4**^2−^ (a) and **1**^2−^ (c), prepared by two electron reduction of **4** and **1**, respectively, at −1.4 V recorded at 77 K in *o*-dichlorobenzene solution containing [*n*-Bu_4_N][BF_4_] as supporting electrolyte. Enlarged regions around the central feature of **4**^2−^ (b) and **1**^2−^ (d) show characteristic zero field splitting parameters for the intermolecular (purple) and intramolecular (orange) triplet states.

The EPR spectrum of **4**^2−^ in frozen solution recorded at 77 K shows an intense central feature at *g* = 2.0001 (Figure 4a) indicating that the majority of the molecules exist as two independent doublet (S = 1/2) radicals and suggesting a small singlet-triplet energy gap [29]. The intramolecular triplet biradical (S = 1) of **4**^2−^ is also present with a zero-field splitting parameter (D) of 27.8 G (Figure 4a,b). This value is in the same range (26–29 G) as that observed for other triplet biradicals of pyrrolidine-functionalised C_60_ derivatives in C_60_–bridge–C_60_ triads [15,28] and gives an average distance of 10 Å between the unpaired electrons [16], a distance well within the range predicted by models of **4** (Figure S4b, Supporting Information File 1). The half-field signal corresponding to the triplet state is not observed which is also consistent with previous reports for fulleride based triplets [30]. The presence of an intramolecular triplet would indicate that the distance between the two fulleropyrrolidine units is short enough to allow through-space interaction despite the lack of electronic conjugation between the interacting units. In addition to the intramolecular triplet, a set of inner features is tentatively assigned to an intermolecular (or “powder”) triplet (D = 7.9 G) that may result from the aggregation of **4**^2−^ molecules in the frozen solution however we do not exclude other possible assignments [31].

The frozen solution EPR spectrum of **1**^2−^ displays a central feature at *g* = 2.0003, consistent with that of a doublet biradical (Figure 4c) that is flanked on each side by broad “wings” that we assign to the presence of an intermolecular triplet and give a maximum D value of 9 G which is similar to that observed in the spectrum of **4**^2−^. The same “wings” around the central feature (*g* 2.0000) have been observed in the EPR spectra of the one electron reduced species of the monomer **7** (Figure S7, Supporting Information File 1) and hence may be explained by intermolecular interactions. Also present are small baseline features (Figure 4d) that may represent the outer features of either an intramolecular or intermolecular triplet. We note similar small baseline features in the spectrum of **7^1^**^−^; in this case their assignment to an intramolecular triplet must be excluded. Hence, by changing the linker from oxalate in **4**^2−^ to terephthalate in **1**^2−^ we have either reduced the interaction of the spin centres or significantly perturbed the formation of an intramolecular triplet biradical.

The EPR spectra of the C_70_ containing compounds **6**^2−^ and **8**^1−^ (Figure S8, Supporting Information File 1) in fluid solution recorded at room temperature (Figure S8a, Supporting Information File 1) and particularly in frozen solution recorded at 77 K (Figure S8b, Supporting Information File 1) are significantly different from those observed for the reduced C_60_ containing compounds. The difference is due to the lower symmetry of the C_70_ (*D*_5_*_h_* compared to *I_h_* of the C_60_) which results in an anisotropic spectrum [30]. In addition, due to this asymmetry the spectra are significantly broader which means that any features corresponding to the triplet biradicals will overlap with the main central features and hence are not resolved. This complicates the assignment of the spin states in the C_70_ containing compounds and hence they were not investigated further in this study.

## Conclusion

We have developed a synthetic methodology for a range of linear, soluble fullerene triads where the nature of the fullerene cage and the length of the bridge between the cages can be controlled. Cyclic voltammetry measurements demonstrate the high electron accepting capacity of these molecules, which can accept up to six electrons reversibly, but indicate no interactions between the fullerene cages in the ground state of the triads, regardless of the nature of the fullerene (C_60_ or C_70_) or the length of the bridge (oxalate or terephthalate). The first and second reduction potentials of C_60_ and C_70_ in the asymmetric triads appear to be indistinguishable, whilst the third reduction of the two fullerene cages is observed as two separate one-electron processes with the reduction potential being slightly less cathodic for the C_70_ cage. EPR spectroscopy measurements of the two electron reduced triads reveal that the nature of the intramolecular electron spin–spin interactions is dependent on the length of the bridge. Specifically, the two electron reduced oxalate bridged triad, where the fullerene cages are separated by a minimum distance of 12 Å, can exhibit strong intramolecular spin coupling with a D value of 27.8 G. Under the same conditions the triad with the terephthalate bridge, where fullerene cages are separated by a minimum distance of over 16 Å, does not show similar strong intramolecular spin coupling and may exist mainly as an independent doublet biradical. Our methodology enables precise control of the inter-fullerene separation thus providing a mechanism for controlling the spin properties of fullerene triads which is important for the future development of molecular electronic and spintronic devices.

## Experimental

C_60_ (99.5%) and C_70_ (95%) were purchased from SES Research and MER corporation respectively. CH_2_Cl_2_ was freshly distilled over CaH_2_ before use. All other reagents and solvents were purchased from Aldrich and used without further purification. Compounds **1**–**3** and **7**–**10** were synthesised according to previously reported procedure [20]. Infra-red spectra were measured as KBr discs using a Nicolet Avatar 380 FTIR spectrometer over the range 400–4000 cm^−1^. ^1^H and ^13^C NMR spectra were obtained using Bruker DPX 300, Bruker DPX 400, Bruker AV(III) 400 or Bruker AV(III) 500 spectrometers. Mass spectrometry was carried out using a Bruker microTOF spectrometer and a Bruker ultraFlexIII MALDI–TOF spectrometer using trans-2-[3-(4-*tert*-butylphenyl)-2-methyl-2-propenylidene]malononitrile (DCTB) as supporting matrix. UV–vis spectra were measured using a Lambda 25 Perkin Elmer Spectrometer. EPR spectra were obtained on a Bruker EMX EPR spectrometer.

### Cyclic voltammetry

Cyclic voltammetric studies were carried out using an Autolab PGSTAT20 potentiostat, using a three-electrode arrangement in a single compartment cell. A glassy carbon working electrode, a Pt wire secondary electrode and a saturated calomel reference electrode (chemically isolated from the test solution via a bridge tube containing electrolyte solution and fitted with a porous Vycor frit) were used in the cell. Experiments were performed under an atmosphere of argon and in anhydrous solvents. Sample solutions were prepared under an atmosphere of argon using Schlenk line techniques and consisted of a 0.2 M [*n*-Bu_4_N][BF_4_] solution as the supporting electrolyte and a 0.5 mM solution of the test compound. Redox potentials were referenced vs the Fc^+^/Fc couple, which was used as an internal standard. Compensation for internal resistance was not applied.

### Bulk electrolysis

Bulk electrolysis experiments at a controlled potential were carried out using a two-compartment cell. A Pt/Rh gauze basket working electrode was separated from a wound Pt/Rh gauze secondary electrode by a glass frit. A saturated calomel electrode was bridged to the test solution through a Vycor frit that was orientated at the centre of the working electrode. The working electrode compartment was fitted with a magnetic stirrer bar and the test solution was stirred rapidly during electrolysis. Each solution contained [*n*-Bu_4_N][BF_4_] (0.2 M) as the supporting electrolyte and the compound under investigation (5 mL, 0.5 mM) and were prepared using Schlenk line techniques.

### Synthesis of the fullerene triads **4**–**6**

#### Synthesis of **4**

[60]Fulleropyrrolidine **9** (35 mg, 0.045 mmol) was suspended in freshly distilled CH_2_Cl_2_ (35 mL) with 4-dimethylaminopyridine (17 mg, 0.139 mmol) and pyridine (0.35 mL), and the reaction mixture was stirred for 10 min. Oxalyl chloride (0.1 mL, 1.16 mmol) was then added, and the reaction mixture stirred at room temperature for 2 h. On removal of solvent, the resulting residue was dissolved in CS_2_ (40 mL) by sonication, and any undissolved material removed by filtration. The filtrate was concentrated and purified by column chromatography (silica gel, *o*-dichlorobenzene/isopropyl alcohol 99.5:0.5) to give the product which was washed with MeOH (30 mL), petroleum ether 40–60 (30 mL) and diethyl ether (30 mL). The resultant solid was dried under vacuum to give the product (16 mg, 44%) as a brown solid. ^1^H NMR (400 MHz, 297 K, CS_2_/CDCl_3_, δ, ppm) 5.79 (s, 4H, CH_2_), 5.69 (s, 4H, CH_2_); ^13^C NMR (125 MHz, 297 K, CS_2_/CDCl_3_, δ, ppm) 160.97 (CO), 153.09, 152.74, 147.47, 146.46, 146.44, 146.26, 146.24, 145.80, 145.72, 145.46, 145.43, 145.40, 145.38, 144.59, 144.46, 143.21, 142.83, 142.80, 142.30, 142.23, 142.21, 142.16, 142.07, 142.05, 140.50, 140.40, 135.97, 135.76, 133.02, 130.61, 128.44, 127.69 (sp^2^ carbons, 31 environments), 70.85, 69.31, 60.22, 57.07 (sp^3^ carbons, 4 environments); MALDI–TOF MS (DCTB/MeCN, *m/z*): 1580.2 (M^−^); IR (KBr, ν, cm^−1^): 2960 (w), 2922 (w), 1645 (s), 1453 (w), 1328 (m), 1186 (m), 746 (m), 526 (s); UV–vis (CS_2_): λ_max_ (ε × 10^−3^/dm^3^ mol^−1^ cm^−1^): 703 (0.750), 433 (8.526).

#### Synthesis of **6**

[70]Fulleropyrrolidine **10** (20 mg, 0.023 mmol) was suspended in CH_2_Cl_2_ (18 mL), and DMAP (8.7 mg, 0.071 mmol) and pyridine (0.2 mL) were added. The reaction mixture was stirred for 10 min and oxalyl chloride (0.055 mL, 0.64 mmol) was added. The reaction mixture was stirred at room temperature for 2 h, and the solvent was then removed. The resulting residue dissolved in CS_2_ (40 mL) by sonication, and the undissolved material removed by filtration. The filtrate was concentrated and purified by column chromatography (silica gel, *o*-dichlorobenzene/isopropyl alcohol 99.5:0.5) to give the product which was washed with MeOH (30 mL) and petroleum ether 40–60 (30 mL). The resultant material was dried under vacuum to give the product (9 mg, 22%) as a brown solid. ^1^H NMR (400 MHz, 297 K, CS_2_/CDCl_3_, δ, ppm) 5.25–4.36 (m, 8H, CH_2_); ^13^C NMR (125 MHz, 297 K, CS_2_/CDCl_3_, δ, ppm) 168.90 (C=O), 167.43 (C=O), 156.55, 156.13, 155.02, 154.96, 154.11, 152.17, 151.56, 151.42, 151.35, 151.07, 151.01, 151.00, 150.96, 150.94, 150.78, 150.71, 150.41, 150.37, 149.93, 149.89, 149.85, 149.79, 149.47, 149.40, 149.36, 149.31, 149.26, 149.11, 149.07, 149.03, 148.79, 148.45, 148.10, 148.06, 147.88, 147.46, 147.21, 147.10, 147.08, 147.05, 147.00, 146.96, 146.93, 146.64, 146.62, 146.57, 145.84, 145.71, 145.53, 145.41, 145.00, 144.90, 144.84, 144.50, 144.48, 144.23, 144.20, 144.16, 144.12, 144.08, 143.48, 143.43, 143.32, 143.26, 143.18, 143.10, 141.43, 140.32, 140.30, 140.27, 137.28, 133.76, 133.72, 133.68, 132.41, 132.13, 131.38, 131.28, 131.25, 128.40 (sp^2^ carbons, 80 environments), 71.84, 69.68, 68.74, 64.10, 62.67, 62.63 (sp^3^ carbons, 6 environments); MALDI–TOF MS (DCTB/MeCN, *m/z*): 1820.5; IR (KBr, ν, cm^−1^): 2932 (m), 2363 (s), 1663 (s, C=O), 1435 (m), 669 (s); UV–vis (CS_2_): λ_max_ (ε × 10^−3^/dm^3^ mol^−1^ cm^−1^): 693 (3.74), 556 (20.32), 476 (39.28), 462 (39.60), 411 (48.49).

#### Synthesis of **5**

Compound **15** (16 mg, 0.018 mmol), [70]fulleropyrrolidine **10** (16 mg, 0.019 mmol) and dicyclohexylcarbodiimide (3.9 mg, 0.019 mmol) were suspended in anhydrous *o*-dichlorobenzene (2.7 mL) and stirred at room temperature under an Ar atmosphere for 17 h. The reaction mixture was purified by column chromatography (silica gel, *o*-dichlorobenzene) to afford the product which was washed with MeOH (30 mL) and petroleum ether (30 mL) to give the product (9 mg, 27%) as a dark brown solid. Isomer **a** (see Supporting Information File 1): ^1^H NMR (400 MHz, 297 K, CS_2_/CDCl_3_, δ, ppm) 5.47 (s, 4H, CH_2_), 5.28 (m, 2H, CH_2_), 4.77 (s, 2H, CH_2_), 4.66 (s, 2H, CH_2_); Isomer **b** (see Supporting Information File 1): ^1^H NMR (400 MHz, 297 K, CS_2_/CDCl_3_, δ, ppm) 5.63 (s, 1H), 4.97 (m, 1H), 4.88 (s, 1H), 4.61 (m, 2H), 4.27 (s, 2H), 4.16 (s, 1H), 4.11 (m, 2H); ^13^C NMR (125 MHz, 297 K, CS_2_/CDCl_3_, δ, ppm) 165.69 (C=O), 155.45, 155.16, 155.07, 154.23, 153.30, 153.19, 151.74, 151.38, 151.21, 151.19, 150.92, 150.79, 150.74, 150.66, 149.91, 149.89, 149.81, 149.36, 149.35, 149.32, 149.24, 149.09, 148.78, 148.44, 148.13, 147.61, 147.41, 147.21, 146.94, 146.36, 146.18, 146.15, 145.94, 145.89, 145.57, 145.40, 145.31, 144.64, 144.60, 143.45, 143.22, 143.07, 142.78, 142.18, 142.03, 140.60, 140.38, 139.15, 137.17, 134.25, 133.75, 132.92 (sp^2^ carbons, 52 environments), 74.22, 73.26, 70.50, 68.18, 68.14, 64.51, 63.11 (sp^3^ carbons, 7 environments); MALDI–TOF MS (DCTB/MeCN, *m/z*): 1686.3; IR (KBr, ν, cm^−1^): 2926 (s), 2365 (s), 1674 (s, C=O), 1433 (m), 1250 (m), 1182 (m), 1119 (m), 671 (w), 527 (m); UV–vis (CS_2_): λ_max_ (ε × 10^−3^/dm^3^ mol^−1^ cm^−1^): 693 (2.51), 556 (11.83), 479 (22.00), 456 (22.91), 430 (22.48), 410 (30.38).

### Oxalic acid monobenzyl ester monochloride [22]

Oxalyl chloride (1 mL) was cooled to 0 °C, and anhydrous benzyl alcohol (1.4 mL) added dropwise over 15 min. After the addition of the alcohol was completed, the reaction mixture was warmed up to room temperature and stirred for 1.5 h. The resulting mixture was analysed by ^1^H and ^13^C NMR spectroscopy and found to be a mixture of the oxalic acid monobenzyl ester monochloride and the dibenzyl oxalate in a 5:1 molar ratio. The mixture was used in the next step immediately without further purification. ^1^H NMR (300 MHz, 297 K, CDCl_3_, δ, ppm) 7.47–7.42 (m, 5H), 7.42–7.38 (m, 1.75 H), 5.38 (s, 2H), 5.32 (s, 0.72 H); ^13^C NMR (75 MHz, 297 K, CDCl_3_, δ, ppm) 160.95, 157.57, 155.53, 134.18, 133.33, 129.35, 128.91, 128.78, 128.74, 70.38, 68.63.

### [60]Fulleropyrrolidine oxalate benzyl ester **11**

[60]Fulleropyrrolidine **9** (70 mg, 0.092 mmol) was suspended in freshly distilled CH_2_Cl_2_ and DMAP (50 mg, 0.41 mmol) and pyridine (0.3 mL) added. The mixture was stirred for 10 min at room temperature, and oxalyc acid monobenzyl ester monochloride (100 mg, 0.50 mmol) was added, and the mixture left to stir at room temperature for 2 h. The solvent was removed, the resulting residue dissolved in CS_2_ (10 mL) and filtered to remove insoluble materials. The filrate was then concentrated and purified by column chromatography (silica gel, toluene) to afford the product which was washed with MeOH (40 mL) and petroleum ether (40 mL) and dried in vacuum to give compound **11** as black powder (58 mg, 68%). ^1^H NMR (400 MHz, 297 K, CDCl_3_/CS_2_, δ, ppm) 7.47 (d, *J* = 6.3 Hz, 2H), 7.35 (m, 3H), 5.51 (s, 2H), 5.45 (s, 4H); ^13^C NMR (125 MHz, 297 K, CDCl_3_/CS_2_, δ, ppm) 160.85 (C=O), 157.49 (C=O), 153.01, 152.57, 147.50, 147.44, 146.49, 146.44, 146.26, 146.24, 145.80, 145.76, 145.61, 145.46, 145.45, 145.43, 145.31, 144.62, 144.51, 143.22, 142.83, 142.82, 142.25, 142.18, 142.10, 142.03, 140.46, 140.32, 137.51, 136.25, 135.94, 134.60, 129.15, 128.99, 128.96, 128.66, 128.44, 128.42, 125.53 (sp^2^ carbons, 37 environments), 70.67, 69.09, 67.99, 59.35, 57.16 (sp^3^ carbons, 5 environments); MALDI–TOF MS (DCTB/MeCN, *m*/*z*): 924.1 (M^−^); IR (KBr, *ν*, cm^−1^): 2924 (m), 2362 (m), 1718 (s, C=O), 1671 (s, C=O), 1438 (m), 1125 (s), 527 (s).

### Synthesis of **12**

Compound **11** (5 mg) was dissolved in freshly distilled CH_2_Cl_2_ (5 mL), and CF_3_SO_3_H (0.05 mL) added. The resulting mixture was stirred for 1 h at room temperature after which the solvent was removed under vacuum and the resultant brown solid was suspended in diethyl ether (10 mL). The precipitate was separated by centrifugation, the ether removed by decantation, and this procedure was repeated three times. The resultant brown solid was dried under vacuum to give the product, **12** (4 mg, 95%) as a brown solid. MALDI–TOF MS (DCTB/MeCN, *m*/*z*): 791.2 (M^−^); IR (KBr, *ν*, cm^−1^): 3446 (s, NH), 2964 (m), 2360 (m), 1636 (s, C=O), 1507 (m), 1384 (s), 1216 (m), 527 (m).

### Synthesis of **13** [24]

NaI (5.3 g, 34 mmol) was suspended in acetone (15 mL) and heated under reflux for 5 min. The mixture was cooled to room temperature, and benzyl bromoacetate (1 mL, 6.3 mmol) added. The reaction mixture was stirred at room temperature for 2 h and the solvent removed under vacuum. The resulting mixture was partitioned between water (20 mL) and ethyl acetate (10 mL). The organic fraction was separated, washed with a saturated solution of Na_2_S_2_O_3_ (2 × 10 mL) followed by brine (10 mL) and dried over Na_2_SO_4_. The solvent was removed to give the product (1.57 g, 90%) as a yellow oil; ^1^H NMR (300 MHz, 297 K, CDCl_3_, δ, ppm) 7.40 (s, 5H), 5.20 (s, 2H), 3.76 (s, 2H); ^13^C NMR (75 MHz, 297 K, CDCl_3_, δ, ppm) 168.60 (C=O), 135.14, 128.65, 128.54, 128.33, 67.81 (-CH_2_-O-), -5.51 (-CH_2_I); ESIMS (MeOH, *m*/*z*): 298.95 (M + Na)^+^.

### Synthesis of **14**

To a solution of [60]fulleropyrrolidine **9** (110 mg, 0.144 mmol) in dry DMF (30 mL) benzyliodoacetate (150 mg) was added, and the resulting mixture heated to 100 °C for 1 h. The solvent was then removed under vacuum, and the resulting solid purified by column chromatography (silica gel, eluted with CS_2_, followed by CS_2_/toluene 1:1 v/v). The product was washed with MeOH (40 mL) and the resultant solid was dried in vacuum to give compound **14** as a black powder (80 mg, 61%). ^1^H NMR (400 MHz, 297 K, CDCl_3_/CS_2_, δ, ppm) 7.49 (d, *J* = 6.8 Hz, 2H), 7.42 (m, 3H), 5.37 (s, 2H), 4.68 (s, 2H), 4.09 (s, 2H); ^13^C NMR (125 MHz, 297 K, CDCl_3_/CS_2_, δ, ppm) 169.31 (C=O), 154.61, 147.37, 146.33, 146.14, 146.03, 145.74, 145.56, 145.36, 144.62, 143.18, 142.72, 142.28, 142.16, 141.98, 140.26, 136.39, 135.64, 135.22, 128.81, 128.62, 128.53, 128.37 (sp^2^ carbons, 22 environments), 70.58, 67.05, 66.78, 54.84 (sp^3^ carbons, 4 environments); MALDI–TOF MS (DCTB/MeCN *m/z*): 911.5 (M^−^); IR (KBr, *ν*, cm^−1^): 2962 (w), 2359 (w), 1736 (s, C=O), 1393 (m), 1344 (m), 1095 (s), 737 (m), 527 (s).

### Synthesis of **15**

To a solution of **14** (5 mg, 0.0055 mmol) in dry CH_2_Cl_2_ (5 mL) CF_3_SO_3_H (0.05 mL) was added and the mixture was stirred for 2 h at room temperature. The solvent was removed under vacuum and the resultant brown solid suspended in diethyl ether (10 mL). The precipitate was separated by centrifugation, the ether removed, and this procedure was repeated three times. The resultant brown solid was dried in vacuum to give the product, **15** (4.2 mg, 90%); MALDI–TOF MS (DCTB/MeCN, *m/z*): 821.2 (M^−^); IR (KBr, *ν*, cm^−1^): 3446 (s, OH), 2957 (w), 2361 (w), 1732 (m, C=O), 1483 (m), 1170 (m), 746 (m), 527 (s).

## Supporting Information

File 1 Additional spectra.
