# Supplementary material for: Tuning the interactions between electron spins in fullerene-based triad systems
Source: Beilstein J Org Chem. 2014 Feb 5;10:332–43. doi: 10.3762/bjoc.10.31 (PMC3943482; doi:10.3762/bjoc.10.31)
Supplement: File 1 — Additional spectra. [file Beilstein_J_Org_Chem-10-332-s001.pdf]

Supporting Information

for

Tuning the interactions between electron spins in fullerene-based triad systems

Maria A. Lebedeva [1], Thomas W. Chamberlain [1], E. Stephen Davies [1], Bradley E. Thomas [1], Martin Schröder[1] and Andrei N. Khlobystov*[1,2]

Addess: [1]School of Chemistry, University of Nottingham, Nottingham, NG7 2RD, UK and [2]Nottingham Nanoscience & Nanotechnology Centre, University of Nottingham, University Park, Nottingham, NG7 2RD, UK

Email: Andrei N. Khlobystov - andrei.khlobystov@nottingham.ac.uk

*Corresponding author

Additional spectra

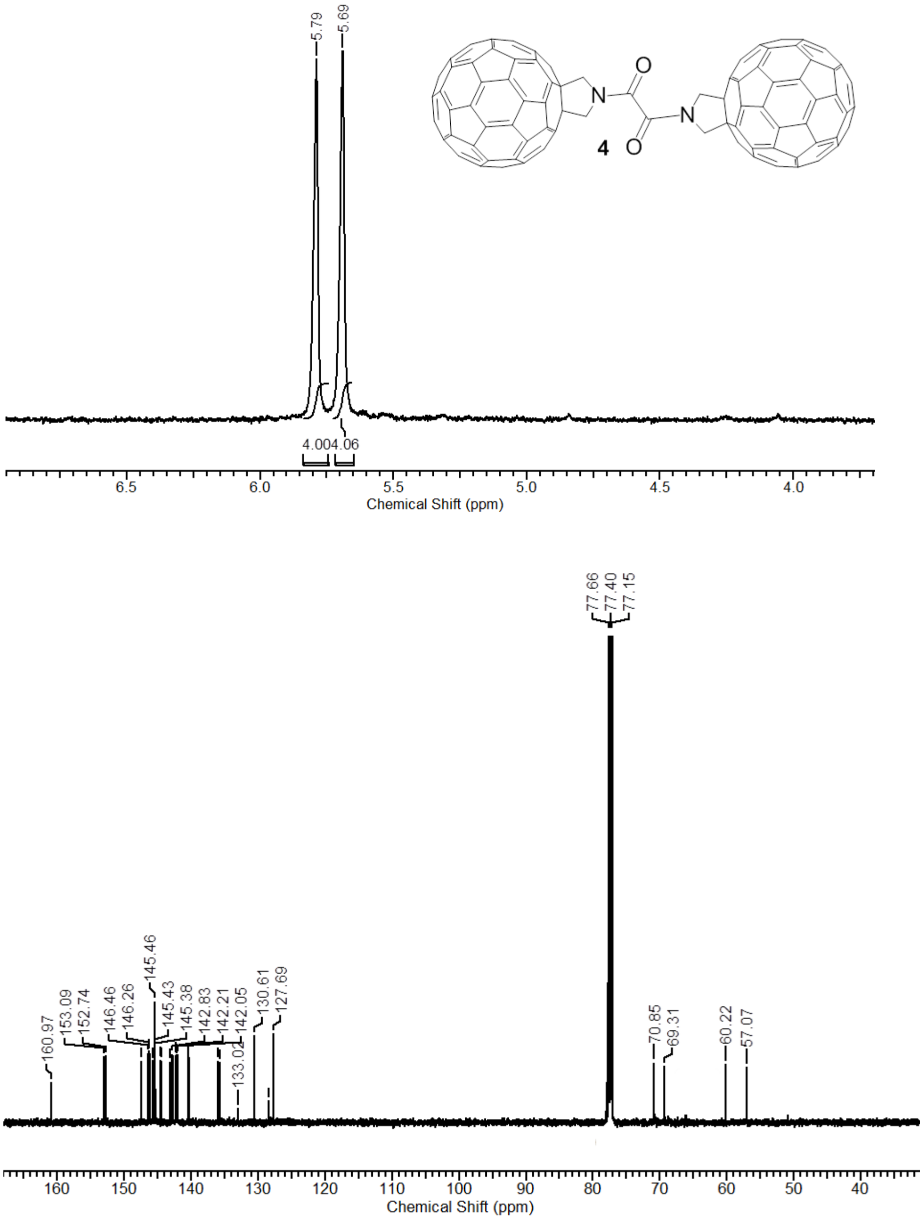

Figure S1: ^{1}H (top) and ^{13}C (bottom) NMR spectra of triad **4**. Spectra were recorded in a 1:1 mixture of CS_2 and $CDCl_3$.

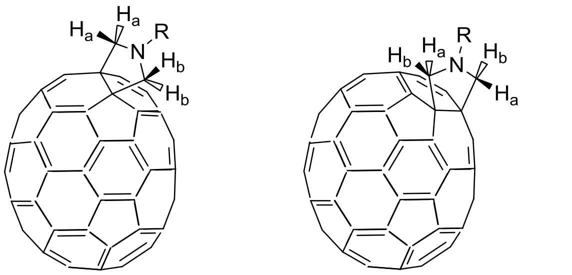

a (8,25)-[70]Fulleropyrrolidine

b (7,27)-[70]Fulleropyrrolidine

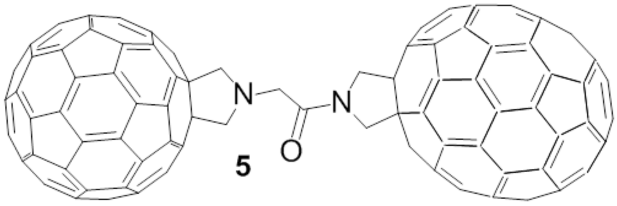

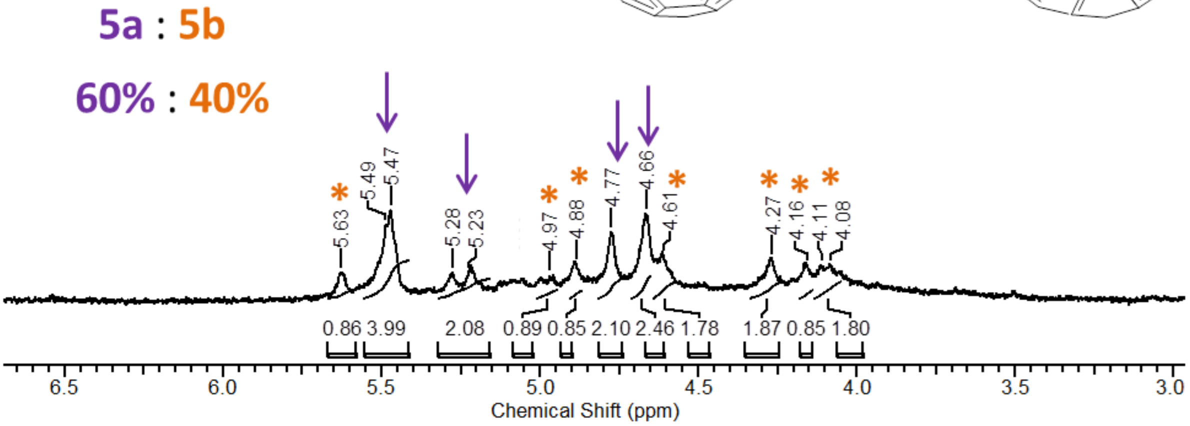

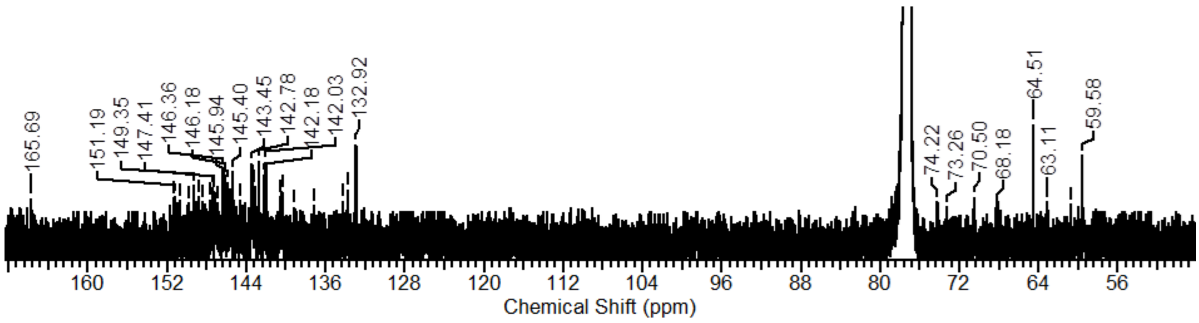

Figure S2: Views of (8,25) (**a**) and (7,22) (**b**) regioisomers of functionalised C_{70} (top) and ^{1}H NMR (middle) and ^{13}C NMR (bottom) spectra of triad **5**. The spectra were recorded in a mixture of CS_2 and $CDCl_3$.

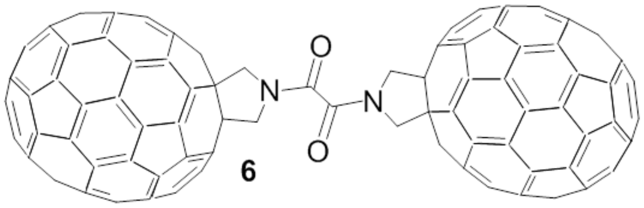

6aa : 6bb : 6ab

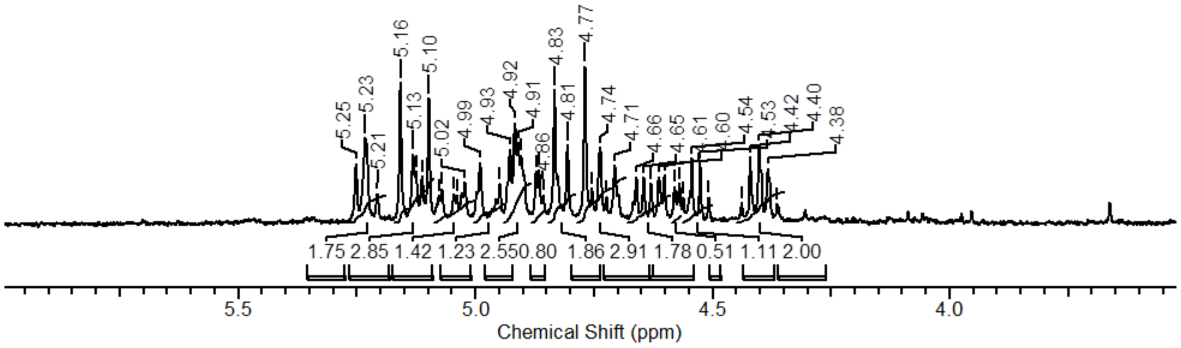

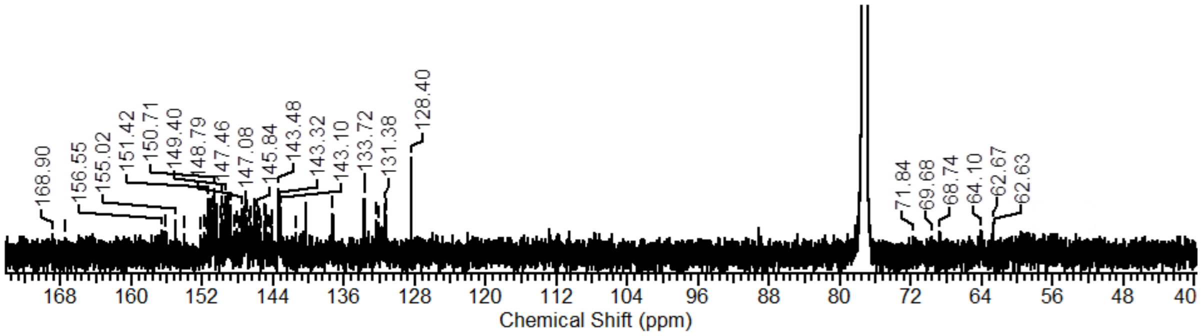

Figure S3: ^{1}H NMR (top) and ^{13}C NMR (bottom) spectra of triad **6**. The spectra were recorded in a mixture of CS_2 and $CDCl_3$.

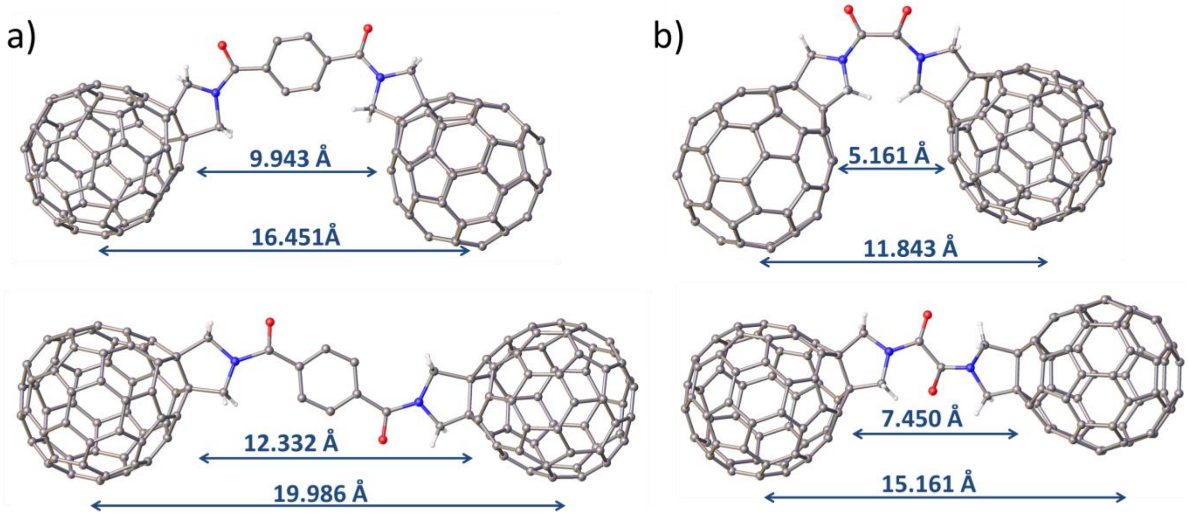

Figure S4: Views of shortest (top) and longest (bottom) possible conformations of fullerene dimers **1** (a) and **4** (b) showing the shortest distances between the fullerene cages and the centre-to-centre distances.

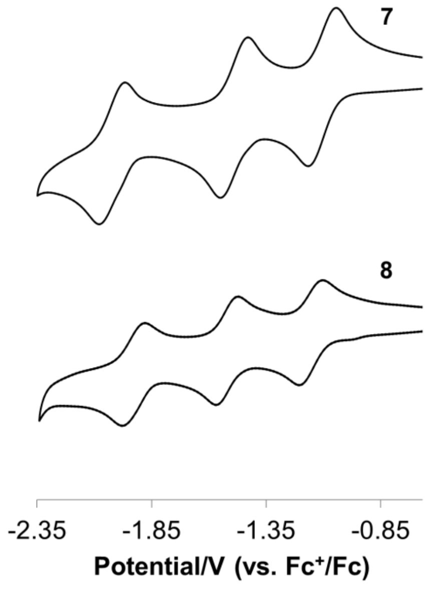

Figure S5: Cyclic voltammograms of **7** (top) and **8** (bottom). Scans were recorded as 0.5 mM solutions in *o*-dichlorobenzene containing 0.2 M [n-Bu_4N][BF_4] as the supporting electrolyte at a scan rate of 100 mV.

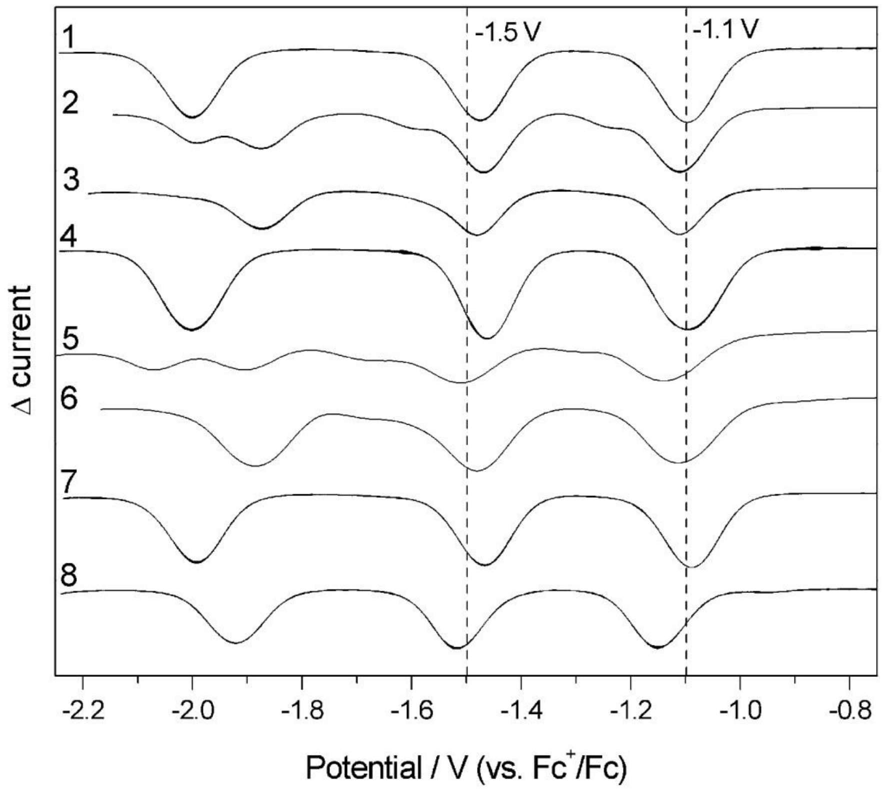

Figure S6: Square wave voltammograms of **1–8**. Scans were recorded as 0.5 mM solutions in *o*-dichlorobenzene containing 0.2 M [*n*-Bu_4N][BF_4] as the supporting electrolyte.

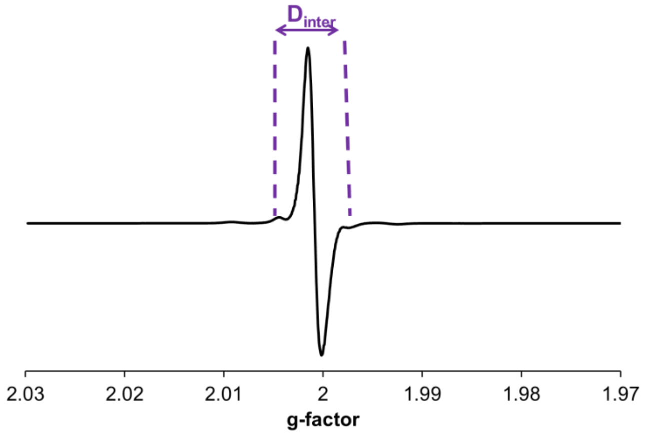

Figure S7: Frozen solution EPR spectrum of $\mathbf{7}^{1-}$ recorded at 77 K showing the central feature corresponding to the C_{60}^{-} and features with D = 9 G corresponding to the intermolecular, or "powder" triplet. Additional small features are noted in the spectrum. The origin of these features is unclear but may result from an unidentified triplet (D = 26 G).

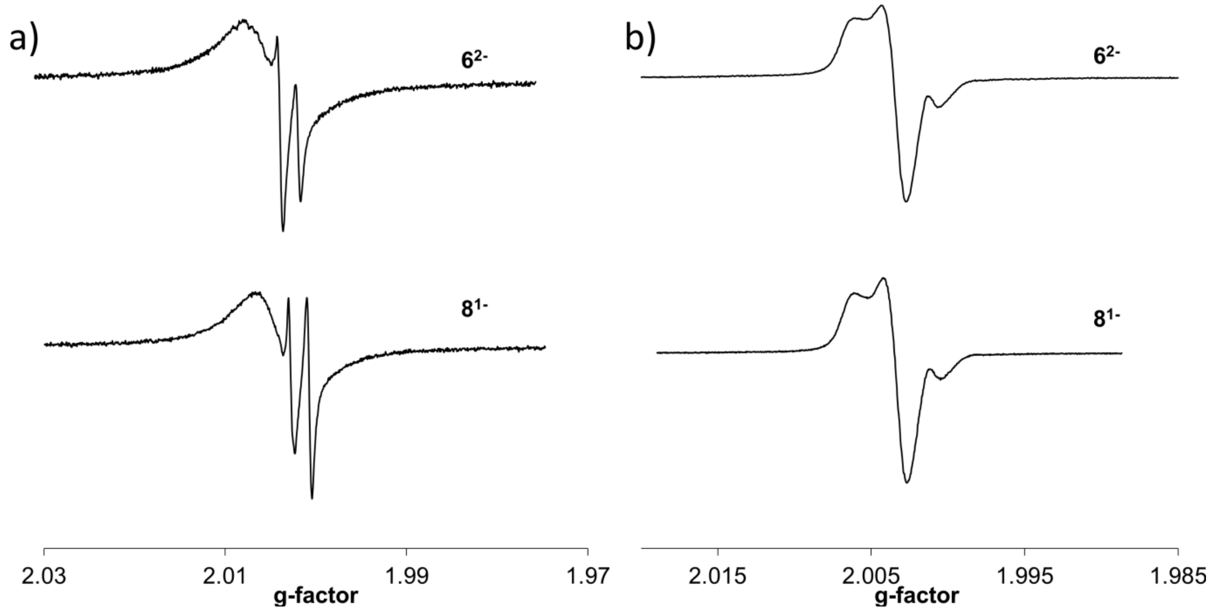

Figure S8: Fluid solution EPR spectra recorded at room temperature (a) and frozen solution EPR spectra recorded at 77 K (b) for $\mathbf{6}^{2-}$ and $\mathbf{8}^{1-}$.
